# Breast Cancer Detection via Multi-Tiered Self-Contrastive Learning in Microwave Radiometric Imaging

**DOI:** 10.3390/diagnostics15050549

**Published:** 2025-02-25

**Authors:** Christoforos Galazis, Huiyi Wu, Igor Goryanin

**Affiliations:** 1 Department of Computing, Imperial College London, London SW7 2AZ, UK; c.galazis20@imperial.ac.uk; 2 National Heart & Lung Institute, Imperial College London, London SW7 2AZ, UK; h.wu21@imperial.ac.uk; 3 School of Informatics, University of Edinburgh, Edinburgh EH8 9YL, UK; 4 Okinawa Institute Science and Technology, Okinawa 904-0412, Japan; 5 MMWR Ltd., Edinburgh EH10 5LZ, UK

**Keywords:** breast cancer detection, microwave imaging radiometry, self-contrastive learning, hierarchical neural networks, point-of-care testing

## Abstract

**Background:** Early and accurate detection of breast cancer is crucial for improving treatment outcomes and survival rates. To achieve this, innovative imaging technologies such as microwave radiometry (MWR)—which measures internal tissue temperature—combined with advanced diagnostic methods like deep learning are essential. **Methods:** To address this need, we propose a hierarchical self-contrastive model for analyzing MWR data, called Joint-MWR (J-MWR). J-MWR focuses on comparing temperature variations within an individual by analyzing corresponding sub-regions of the two breasts, rather than across different samples. This approach enables the detection of subtle thermal abnormalities that may indicate potential issues. **Results:** We evaluated J-MWR on a dataset of 4932 patients, demonstrating improvements over existing MWR-based neural networks and conventional contrastive learning methods. The model achieved a Matthews correlation coefficient of 0.74 ± 0.02, reflecting its robust performance. **Conclusions:** These results emphasize the potential of intra-subject temperature comparison and the use of deep learning to replicate traditional feature extraction techniques, thereby improving accuracy while maintaining high generalizability.

## 1. Introduction

Breast cancer, characterized by the uncontrolled and rapid growth of cells due to genetic mutations, poses a significant global health challenge. It has one of the highest incidence rates among all cancers, with an estimated 2.3 million new cases and nearly 700,000 deaths reported in 2020 alone. These figures underscore its status as the leading cause of cancer-related mortality among women [1]. Alarmingly, projections indicate a continued increase in both the incidence and mortality rates of breast cancer in the coming years [2].

Early detection plays a pivotal role in reducing mortality rates and alleviating the healthcare burden. In this context, microwave imaging radiometry (MWR) has emerged as a promising imaging modality. MWR passively captures the natural microwave emissions from human tissues, offering a versatile diagnostic tool [3]. Its applications extend across a wide range of clinical domains, including breast imaging [3,4,5,6,7,8], brain temperature monitoring [9,10,11,12], lung diagnostics [13,14], vein assessment [15], and musculoskeletal evaluations [16,17].

In cancer diagnostics, MWR leverages the increased metabolic activity of cancerous tissues, which emit higher levels of heat compared to healthy tissues [4]. Compared to traditional screening methods, MWR offers several distinct advantages, including its non-invasive nature, safety, portability, and cost-effectiveness [3]. However, the relatively recent introduction of MWR in breast cancer diagnostics presents challenges, particularly in data interpretation and the integration of this technology into existing medical workflows. To address these challenges, the development of advanced artificial intelligence models is essential to optimize and streamline the clinical applications of MWR.

In recent years, substantial progress has been made in leveraging machine learning and deep learning techniques to improve the diagnostic accuracy of MWR-based breast cancer detection. Traditional machine learning methods, such as support vector machines (SVMs), random forests, and Bayesian classifiers, have demonstrated notable efficacy in this domain [18,19,20]. Additionally, neural-network-based approaches have shown considerable promise [18,19,21,22]. For example, advancements include the development of learnable architectures designed for lightweight and resource-efficient neural networks [23], as well as the integration of Kohonen’s self-organizing maps with machine learning algorithms [6]. Furthermore, fuzzy analysis techniques have played an important role in improving system efficacy [24,25].

Before the adoption of data-driven methods, hand-engineered features were developed based on expert knowledge to identify temperature asymmetries within the breast [5,26,27]. These features can be categorized into five groups: (1) temperature asymmetry between the two glands; (2) increased temperature dispersion within a single gland; (3) the detection of abnormally high temperatures in the nipple relative to other gland regions; (4) relationships between surface and depth temperature measurements; and (5) features derived from a comparative analysis of the two glands.

Despite these advancements, integrating domain knowledge into data-driven methodologies remains a significant challenge. Combining hand-engineered features with machine learning models is critical for improving both diagnostic performance and generalizability. These improvements are essential to ensure the clinical applicability of MWR across diverse and evolving scenarios.

**Figure 1:**
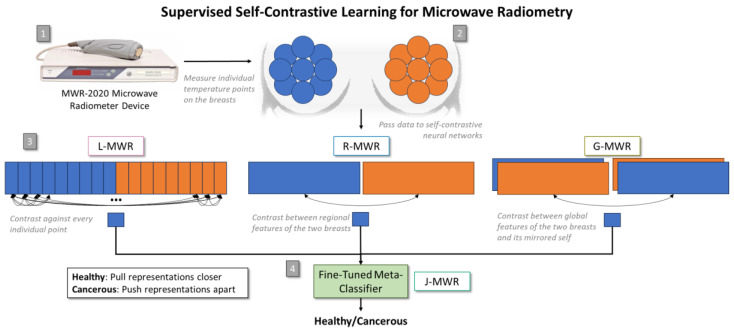
Overview of the proposed self-contrastive models for breast cancer detection. (1) A microwave radiometry (MWR) device, MWR-2020, is used to capture skin and internal temperatures at (2) predetermined locations on the breasts. (3) The data are processed through three hierarchical levels of supervised self-contrastive models: Local-MWR (L-MWR) compares individual temperature points, Regional-MWR (R-MWR) compares temperature features between the left and right breasts, and Global-MWR (G-MWR) compares features of both breasts with their inverted counterparts. (4) The outputs from these pre-trained models are aggregated and fine-tuned using a meta-classifier, Joint-MWR (J-MWR).

This paper introduces a novel supervised neural network, Joint-MWR (J-MWR), which integrates three hierarchical self-contrastive learning networks to improve MWR-based breast cancer detection. As illustrated in Figure 1, J-MWR employs self-contrastive learning to analyze and compare different regions within the same instance, departing from traditional methods that compare data across cases [5,26,27]. By embedding domain knowledge into a data-driven framework, J-MWR bridges the gap between traditional feature extraction techniques and the scalability of neural networks. The experimental results demonstrate that J-MWR outperforms state-of-the-art MWR models and widely used batch-wise contrastive learning methods, underscoring its potential as a robust solution for breast cancer detection.

Traditional contrastive learning [28] optimizes feature representations by enforcing similarity between positive pairs (e.g., augmented views of the same sample) while pushing apart negative pairs (e.g., different samples). This approach relies on comparisons across multiple samples, requiring large and diverse datasets to establish meaningful relationships. While effective, it introduces several challenges, including batch dependence, the need for careful pair selection strategies, and reliance on extensive data augmentation.

In contrast, our proposed self-contrastive learning approach operates within individual cases rather than across multiple samples. Instead of learning from cross-sample relationships, it focuses on refining representations by leveraging internal differences. This inward-looking methodology eliminates the need for extensive sample-pairing strategies and mitigates issues related to false negatives.

In summary, our contributions are as follows:

- To the best of our knowledge, this work represents the first evaluation of MWR data using supervised contrastive learning.
- We propose a novel paradigm that integrates data-driven methodologies with existing knowledge through self-contrastive learning. In this approach, regions within the same subject are compared against each other rather than across samples. We introduce three hierarchical levels of MWR breast self-contrastive models: L-MWR, R-MWR, and G-MWR.
- We developed a meta-classifier, J-MWR, that effectively combines and shares information between the multiple hierarchical self-contrastive models, resulting in improved performance.
- We performed an extensive evaluation of the generalizability of supervised contrastive learning and self-contrastive learning on a large MWR breast dataset.

## 2. Materials and Methods

### 2.1. Data

The dataset used in this study was collected using the MWR-2020 dual-band point-of-care device, developed by MMWR Ltd., UK (https://www.mmwr.co.uk/ (accessed on 1 February 2025). This device monitors both infrared (skin surface temperature) and microwave (internal tissue temperature) emissions. Operating within a microwave frequency range of 3.5 to 4.2 GHz, it is capable of penetrating tissue depths of 3 to 7 cm. Temperature measurements are acquired with an accuracy of ±0.2 °C.

**Figure 2:**
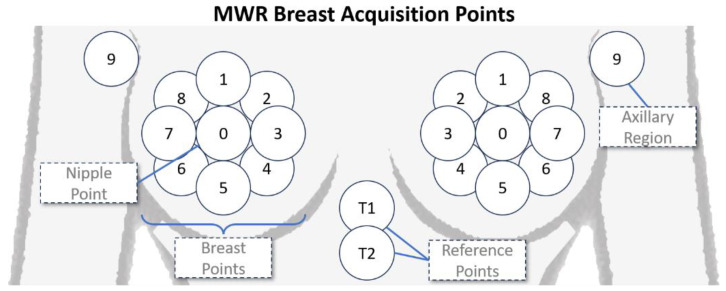
An illustration of the skin and internal acquisition points on the breasts. Point 0 represents the nipple, points 1–8 are arranged equidistantly around the nipple, point 9 corresponds to the axillary region, and reference points T1 and T2 are located beneath the chest.

Data collection involved recording temperatures at 22 specific locations for each case, as depicted in Figure 2. For each breast, eight points were measured equidistantly around the nipple, alongside one measurement directly at the nipple. Additional measurements included the left and right axillary reference regions and two reference points beneath the chest. This resulted in a total of 44 temperature readings (22 skin temperatures and 22 internal temperatures) per case, which served as the input features for the models.

**Figure 3:**
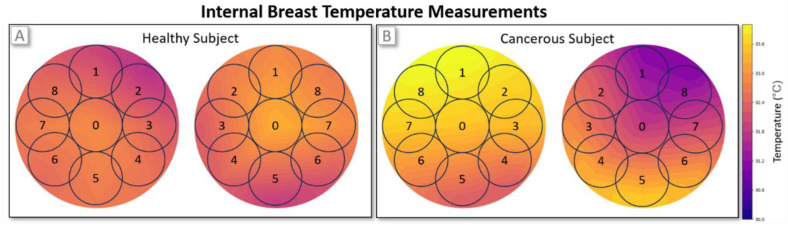
Comparison of internal breast temperature profiles between a healthy case (**A**) and a high-growth-rate cancerous case (**B**). In the healthy case, temperature profiles exhibit no significant asymmetries. On the other hand, in the cancerous case, elevated temperatures are observed in regions 1 and 8 of the right gland, indicating localized abnormalities.

The final dataset consists of measurements from 4932 female cases, collected across multiple clinical centers with full ethical approval and informed consent. Prior to analysis, the data were anonymized, and cases with missing temperature readings were excluded. No additional preprocessing was applied. Of the remaining cases, 4384 were classified as healthy (label = 0) by clinical experts, while 548 were labeled as cancerous (label = 1). Representative examples of a healthy case and a cancerous case are illustrated in Figure 3A and Figure 3B, respectively.

The dataset was randomly split into training (60%), validation (20%), and testing (20%) sets, ensuring class balance was maintained. Model evaluation was performed on the testing set after completing the development and experimentation phases.

### 2.2. Network Architecture

The models in this study were trained under consistent initialization and training settings to ensure comparability. Weights were initialized using the Glorot uniform method [29], and biases were set to zero. The Adam optimizer [30] was used for weight optimization, with an initial learning rate of 0.0001 and parameters β1 = 0.9 and β2 = 0.999. These settings were chosen based on findings from prior studies [18,19] using similar data. To further improve training stability, the learning rate was reduced by a factor of 0.1 if the validation loss did not improve after five consecutive epochs. The batch size was set to 4, determined experimentally as optimal across all models.

To address the class imbalance between healthy and cancerous cases, a class-balanced binary cross-entropy loss function was used. This approach ensured that the models maintained equal focus on both majority (healthy) and minority (cancerous) classes during training.

### 2.3. Base Neural Network

The base model builds upon the current state-of-the-art neural network architecture previously proposed for MWR analysis [18], which was designed for a similar but smaller dataset. To adapt this architecture to the larger dataset used in this study, several modifications were introduced to improve scalability and optimize performance, as described next.

**Figure 4:**
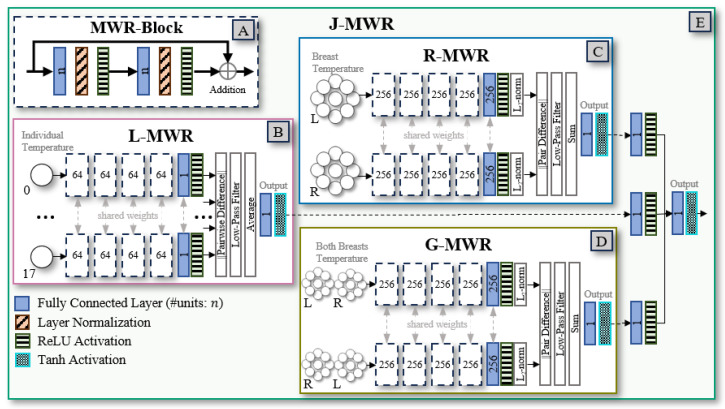
Overview of the proposed multi-tiered self-contrastive MWR models for breast cancer detection. (**A**) The shared MWR-Block, a common residual block used across all networks. (**B**) L-MWR network, which performs point-to-point comparisons within each breast. (**C**) R-MWR network, which conducts comparisons between the left and right breasts. (**D**) G-MWR network, which compares each breast with its positional inverse counterpart. (**E**) J-MWR network, which integrates predictions from L-MWR, R-MWR, and G-MWR to produce the final prediction.

The base model consists of four residual fully connected (FC) modules, referred to as MWR-Blocks, as shown in Figure 4A. Each MWR-Block consists of the following components: an FC layer, layer normalization [31], a Rectified Linear Unit (ReLU) activation function [32], a second FC layer, another layer normalization, a second ReLU activation, and a residual connection that sums the block’s input to its final output.

The optimized base model features four MWR-Blocks, with each FC layer containing 256 units. The output layer is a single-unit FC layer with a sigmoid activation function for binary classification.

### 2.4. Self-Contrastive MWR Neural Networks

Inward learning, not outward wandering: Our proposed self-contrastive models aim to optimize the embedding space by distinguishing features within individual cases, rather than relying on comparisons across multiple samples. This inward-focused approach is aimed at improving predictive accuracy and generalizability.

The proposed J-MWR framework functions as a meta-classifier, integrating three self-contrastive models that operate at different hierarchical levels. This structured approach combines fine-grained, regional, and systemic analyses, positioning J-MWR as a versatile and scalable solution for MWR-based breast cancer screening. During training, information is indirectly shared across tiers, while during inference, their predictions are combined to improve robustness and overall performance.

The simplest tier, Local-MWR (L-MWR), performs point-to-point comparisons across both breasts, enabling detailed intra-breast analysis to detect localized anomalies. This is particularly effective at detecting high-growth-rate tumors.

The second tier, Regional-MWR (R-MWR), extracts more complex, region-dependent features. It compares corresponding regions across the two breasts to detect symmetries or anomalies, capturing cases where the tumors are larger or their growth rates are slowing down.

At the highest level, the Global-MWR (G-MWR) tier utilizes features from both breasts to detect subtle asymmetries that might indicate early signs of cancer. This is achieved by swapping the temperature values of the breasts and comparing them against the original values, leveraging the fact that healthy individuals typically exhibit only minor temperature variations between their breasts.

#### 2.4.1. Local-MWR Neural Network

The L-MWR neural network processes each temperature point individually, excluding reference measurements, resulting in 18 inputs that consist of both skin and internal temperature values (see Figure 4B). The network is divided into two main stages: feature extraction and feature comparison.

In the feature extraction stage, four 64-unit MWR-Blocks are used, followed by a single-unit FC layer with ReLU activation. The weights of the layers are shared across all inputs, ensuring consistent feature learning across the data points.

In the feature comparison stage, the network calculates the absolute differences between features extracted from all pairwise inputs. These differences are filtered by setting values below a learnable threshold to zero, effectively discarding small, noisy variations. This filtering mechanism, experimentally validated, was shown to improve performance across all proposed networks, helping focus on significant discrepancies while reducing sensitivity to noise. Since multiple pairs are processed, the filtered differences are averaged to produce a mean comparison value.

The final prediction is generated using a single-unit FC layer with tanh activation as the estimated mean value is already bounded below zero.

#### 2.4.2. Regional-MWR Neural Network

The R-MWR neural network performs self-contrastive analysis by comparing corresponding regions of the left and right breasts (see Figure 4C). It processes two input vectors, each of size 24, which include breast-specific and reference temperature points. Similar to the L-MWR network, it is structured into two main stages: feature extraction and feature comparison.

In the feature extraction stage, four MWR-Blocks are used, followed by a FC layer with ReLU activation, each containing 256 units. The weights of these layers are shared between the left and right inputs to ensure consistent feature extraction. To prevent extreme values and improve stability, the feature embeddings for each input are normalized using l2-normalization.

In the feature comparison stage, element-wise absolute differences between the normalized feature embeddings of the two inputs are calculated. These differences are filtered by setting values below a learnable threshold to zero, and the remaining values are summed to produce a single comparison score.

The final prediction is generated by a single-unit FC layer with tanh activation.

#### 2.4.3. Global-MWR Neural Network

The G-MWR neural network learns features from both breasts (see Figure 4D). To facilitate self-contrastive learning, G-MWR uses a transformed version of the original input as its second input, where the left and right breast values are swapped (i.e., the left breast values are used as the right, and vice versa). The network takes two pairs of input, each of size 44, and uses the same architecture as the R-MWR network, described in Section 2.4.2.

#### 2.4.4. Joint-MWR Neural Network

The J-MWR meta-classifier combines the pre-trained L-MWR (Section 2.4.1), R-MWR (Section 2.4.2), and G-MWR (Section 2.4.3) models to leverage their complementary strengths (see Figure 4E). Each sub-network’s output is weighted using an individual FC layer and then concatenated into a unified feature vector. This combined vector is passed through a final single-unit FC layer with tanh activation to generate the final prediction. Since only the weights of the sub-networks are fine-tuned, the learning rate is reduced to 1 × 10^−7^ to preserve the learned features while allowing gradual optimization.

### 2.5. Experiments

To ensure robustness and reliability, each model was trained and evaluated over three runs with different random initialization seeds. The results reflect the average performance across these runs on the test set.

Model evaluation was primarily based on Matthews correlation coefficient (MCC), which is particularly useful for assessing performance on imbalanced datasets where both positive and negative cases are equally important [33]. Additionally, accuracy and the area under the receiver operating characteristic curve (ROC AUC) are reported as supplementary metrics.

Comparative analyses were conducted between the proposed self-contrastive models and widely used batch-wise contrastive loss functions, including contrastive loss [34], triplet hard loss [35], triplet semi-hard loss [36], and N-pairs loss [37]. For these configurations, the weight for contrastive losses was experimentally set to 0.1, while cross-entropy classification loss was retained a weight of 1.0.

## 3. Results

### 3.1. Model Evaluation

Our proposed J-MWR model demonstrates the highest predictive capability for identifying breast cancer from MWR data. It achieves an MCC score of 0.74 ± 0.018, outperforming the second-best model, R-MWR, by a margin of 0.08. This corresponds to an accuracy of 0.95 ± 0.003 and an ROC AUC of 0.96 ± 0.001. In comparison, the base model achieves an MCC score of 0.58 ± 0.004, an accuracy of 0.88 ± 0.003, and an ROC AUC of 0.93 ± 0.006. A summary of the results for all models is presented in Table 1.

Among the individual sub-networks, both R-MWR and G-MWR outperform the base model, with MCC scores of 0.66 ± 0.012 and 0.61 ± 0.045, respectively. However, L-MWR underperforms relative to the base model, with an MCC score of 0.43 ± 0.002. This outcome aligns with expectations as L-MWR learns features only from individual points, limiting its predictive power.

### 3.2. Batch-Wise Contrastive Loss Evaluation

When incorporating batch-wise contrastive loss, J-MWR with triplet hard loss achieves the highest performance among the contrastive configurations, with an MCC score of 0.74 ± 0.03, as shown in Table 2. Overall, J-MWR consistently achieves the highest MCC score compared to other models, regardless of the batch-wise contrastive loss used. However, none of the configurations surpass J-MWR without batch-wise contrastive learning.

Interestingly, the impact of batch-wise contrastive learning varies by model. Both R-MWR and J-MWR experience a slight reduction in MCC scores when contrastive losses are applied. In contrast, L-MWR shows a modest improvement of 0.01 in MCC, while G-MWR exhibits a more substantial improvement, with MCC gains ranging from 0.03 to 0.06. Similarly, the base model benefits from applying contrastive and N-pairs losses, achieving a 0.02 increase in MCC for both configurations.

### 3.3. Embedding Space Analysis

**Figure 5:**
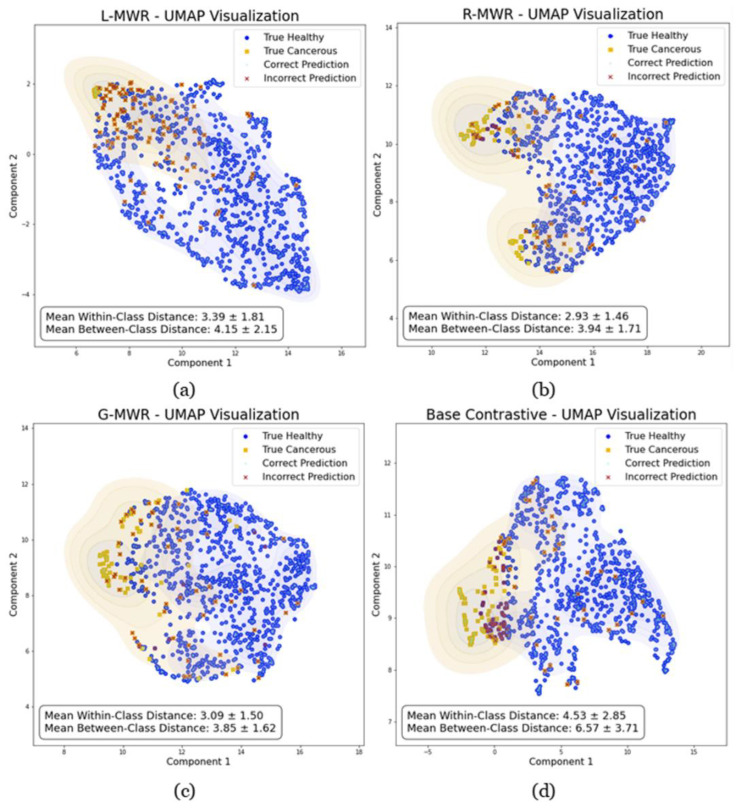
Uniform manifold approximation and projection (UMAP) [35] visualizations of the embedding spaces generated by (**a**) L-MWR, (**b**) R-MWR, (**c**) G-MWR, and (**d**) base contrastive models. In each plot, blue circles represent correct healthy predictions, yellow squares denote correct cancerous predictions, and red crosses overlaid on these markers indicate incorrect predictions.

To investigate the properties of the embedding spaces generated by the L-MWR, R-MWR, G-MWR, and base contrastive models, we used uniform manifold approximation and projection (UMAP) [38] for 2D visualization, as shown in Figure 5. Our analysis reveals that the base contrastive model excels at creating a clearer separation between healthy and cancerous groups, with a mean between-class Euclidean distance of 6.57 ± 3.71. This outcome is expected as the base model is explicitly trained for this delineation. However, it exhibits greater disparities within each group, reflected in a mean within-class distance of 4.53 ± 2.85, contributing to its relatively lower overall performance.

In contrast, our proposed self-contrastive models demonstrate consistent embedding properties for cancerous cases across instances, even though their embeddings are more interwoven with those of healthy cases, making boundary separation less distinct. For example, R-MWR achieves a mean within-class distance of 2.93 ± 1.46 and a mean between-class distance of 3.94 ± 1.17. This closer proximity within groups mitigates the lack of a clear boundary, supporting improved classification performance. These findings suggest that self-contrastive and batch-wise contrastive approaches may be complementary, and their successful integration could further enhance performance.

### 3.4. Data Constraint Training

**Figure 6:**
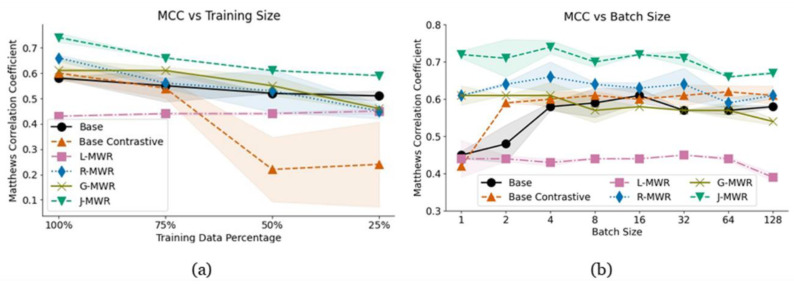
(**a**) Matthews correlation coefficient (MCC) scores for each model when trained on reduced datasets with 75%, 50%, and 25% of the original training size. (**b**) MCC scores for the models across varying batch sizes, ranging from 1 to 128.

To evaluate model scalability with limited training data, we retrained the models using randomly selected subsets of the training set at 75%, 50%, and 25% of the original size. This analysis provides insights into how model performance adapts to varying data availability. The results are summarized in Figure 6a. J-MWR consistently achieves the highest MCC across all subsets, with its lowest performance recorded at 0.69 ± 0.001 when using only 25% of the data. In contrast, the base contrastive model shows the weakest performance when 50% or less of the training data are used, but it noticeably improves at 75%.

Interestingly, L-MWR maintains a stable yet low performance level regardless of the reduction in training data size, reflecting its limited reliance on larger datasets. R-MWR, on the other hand, achieves the highest performance improvement, with a 0.1 increase in MCC when training data are expanded from 75% to 100%. This trend is also reflected in J-MWR, which depends on R-MWR, though the impact is less pronounced. If this trend continues with even larger datasets, R-MWR has the potential to surpass the combined performance of J-MWR.

For the other models, performance tends to plateau as more data are added, suggesting diminishing returns from larger datasets for these configurations.

### 3.5. Batch Size Dependency

We analyze the effect of batch size on performance across values ranging from 1 to 128. J-MWR consistently outperforms all other models, achieving its highest MCC score of 0.74 ± 0.018 at a batch size of 4. Beyond this point, performance gradually declines, as illustrated in Figure 6b. Overall, the proposed self-contrastive models show reduced performance as the batch size increases.

In contrast, the base contrastive model, followed by the base model, maintains a more consistent performance after a batch size of 4. We anticipate this trend will extend to larger batch sizes. This stability highlights a significant distinction in the behavior of batch-wise contrastive losses compared to self-contrastive models.

For self-contrastive models, smaller batch sizes are optimal for accuracy but come at the cost of longer training times. On the other hand, batch-wise contrastive approaches for MWR show minimal dependency on batch size, making them more efficient for scenarios requiring larger batches or faster training.

### 3.6. Generalizability

**Figure 7:**
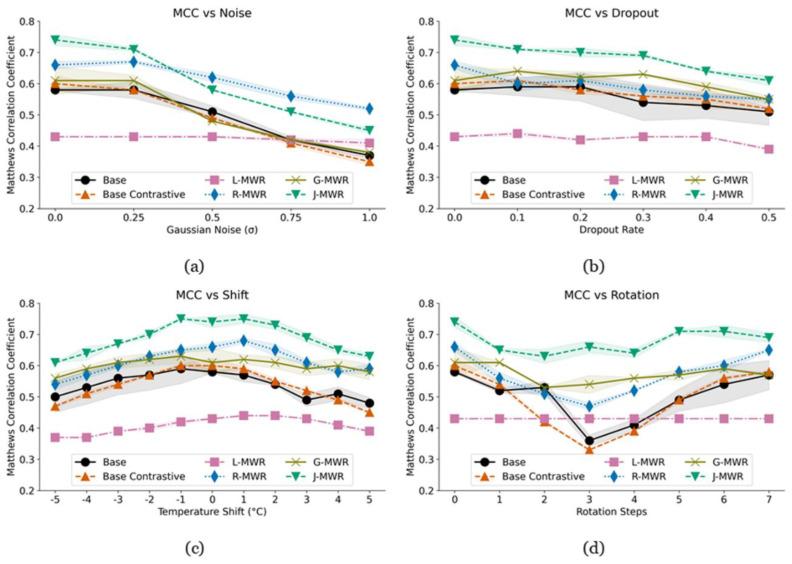
Matthews correlation coefficient (MCC) scores of the models under various data augmentations: (**a**) adding increasing levels of Gaussian noise, (**b**) randomly setting points to the mean of the remaining points, (**c**) uniformly shifting all temperatures by a given amount, and (**d**) rotating the points around the nipple for each breast.

To evaluate the generalizability of our trained models, we tested them on an augmented dataset featuring a range of out-of-distribution transformations. These include the addition of Gaussian noise (Figure 7a), applying dropout to temperature points by replacing their values with the mean of the remaining points (Figure 7b), global temperature shifts across all values (Figure 7c), and rotations of breast points around the nipple (Figure 7d).

As shown in Figure 7, J-MWR consistently demonstrates better generalization performance, maintaining a higher MCC score across various transformations. This holds for both data corruptions (Figure 7a,b) and data drifts (Figure 7c,d). However, under severe Gaussian noise (σ > 0.25), R-MWR exhibits greater resilience as J-MWR’s performance is limited by the degraded performance of G-MWR under noisy conditions.

While L-MWR shows the least variation across transformations, its MCC remains low at approximately 0.43. Interestingly, under certain conditions (see Figure 7), it outperforms the base, base contrastive, and/or G-MWR models.

Overall, these results highlight the robustness of J-MWR in handling diverse data augmentations, albeit with some sensitivity to specific noise types. Its ability to generalize to unknown distributions stems from its focus on comparing regions within individual cases rather than relying on cross-sample comparisons or the training distribution.

### 3.7. Ensemble Methods

**Figure 8:**
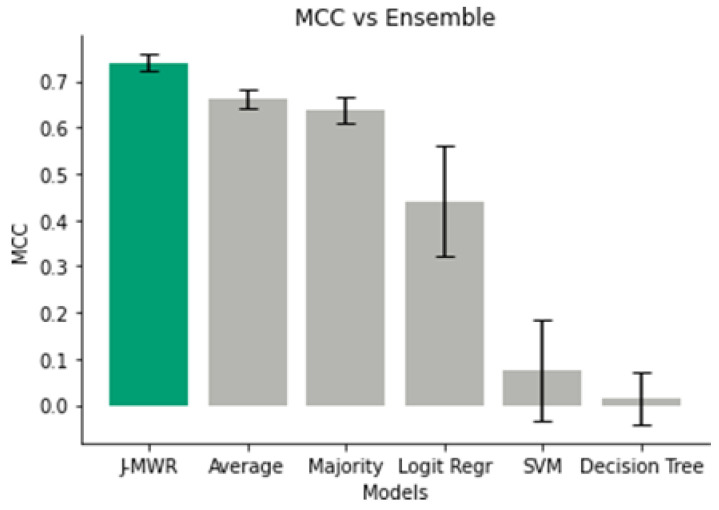
Matthews correlation coefficient (MCC) scores for ensemble methods, including J-MWR, averaging, majority voting, logistic regression, support vector machine (SVM), and decision tree, leveraging the pre-trained L-MWR, R-MWR, and G-MWR models.

Each sub-network in the J-MWR meta-classifier contributes nearly equally to the final prediction. The L-MWR has a contribution weight of 0.998, comparable to R-MWR and G-MWR, which both have weights much closer to 1.0. Additionally, the weight biases of the sub-networks are close to zero. As illustrated in Figure 8, J-MWR consistently outperforms alternative ensemble techniques, including averaging and majority voting, as well as meta-classifiers such as logistic regression, SVM, and decision trees.

While J-MWR bears a resemblance to average voting, given the previous weights, the fine-tuning applied to its sub-networks significantly boosts MCC performance. Averaging the predictions of L-MWR, R-MWR, and G-MWR achieves the second-best MCC score of 0.66 ± 0.02, which aligns closely with the performance of R-MWR alone. Majority voting follows closely behind in performance.

In contrast, meta-classifiers such as logistic regression, SVM, and decision trees demonstrate overfitting during training, leading to large drops in MCC performance on the test set. These results emphasize the effectiveness of J-MWR in leveraging its fine-tuned sub-networks to deliver improved predictive capabilities compared to traditional ensemble methods.

## 4. Discussion

This study successfully demonstrates how self-contrastive learning can improve the performance of MWR breast cancer detection. The proposed architectures, L-MWR, R-MWR, and G-MWR, integrate hand-engineered features capturing thermal asymmetries with data-driven learning. Our meta-classifier, J-MWR, combines the outputs of these individual models and achieves an MCC score of 0.74 ± 0.018, outperforming all existing models. These results underscore the effectiveness of self-contrastive learning in improving both accuracy and generalizability for breast cancer detection, positioning it as a promising advancement in the field.

The final layer of J-MWR effectively functions as an ensemble averaging mechanism, given the near-equal weights of each sub-network. However, J-MWR achieves an MCC improvement of 0.08 compared to traditional ensemble averaging. This boost stems from its role as a fine-tuning meta-classifier, enabling indirect information sharing across the self-contrastive tiers. While this design architecture boosts performance, it comes with the trade-off of increased model complexity and training time.

Furthermore, J-MWR demonstrates strong generalizability, even when trained on a limited dataset, due to two key factors. First, the individual self-contrastive networks in J-MWR mimic hand-engineered feature extraction, which is inherently robust to out-of-distribution data. Second, by incorporating elements of supervised learning, J-MWR can identify more complex and subtle features that may not be captured by domain experts. This allows it to uncover deeper relationships within the data, improving performance and generalizability.

Batch-wise contrastive learning, while yielding modest improvements, holds potential as a minimally disruptive addition to MWR models. Its current subpar performance can be attributed to the inherent variability in breast temperature readings, which are influenced by factors such as age and menstrual cycle phase [3]. By addressing these nuances, batch-wise contrastive learning could further complement self-contrastive approaches, contributing to the development of more robust diagnostic models as it has shown strong results in other modalities for breast cancer [39,40,41,42]. Additionally, other methods can be utilized, such as transfer learning and explainability techniques, as outlined in Table 3, and compared with our proposed self-contrastive learning.

While self-contrastive learning shows promising results for MWR breast analysis, it has certain limitations. It is expected to be less effective for patients with mastectomies, reconstructive surgeries, or hormonal therapies, which alter thermal profiles and reduce the reliability of asymmetry-based comparisons. Its extension to anatomical regions lacking symmetrical properties is also challenging, limiting its broader applicability.

Another challenge is the manual definition of sub-networks, which can be time-consuming and require specialized domain expertise, making it more complex than traditional supervised learning methods. The increased model complexity may also pose challenges for deployment on resource-constrained devices. However, since the MWR-2020 device collects sparse data points, standard clinical hardware should remain sufficient for running the model and within the existing software. Addressing these limitations will be crucial for improving the versatility and accessibility of MWR self-contrastive learning frameworks, especially in point-of-care settings.

Future work will explore the integration of representation learning methods [43] and the use of neural architecture search to automate sub-network design [23], reducing reliance on domain expertise. Alternatively, to simplify the model, we will investigate knowledge distillation [44], a technique that transfers knowledge from multiple sub-networks into a single, simpler model. Additionally, we will evaluate transformer networks [45] as their patch-based processing aligns closely with the regional self-contrastive analysis used in our framework, potentially alleviating the need for defining sub-networks.

Furthermore, we plan to integrate multi-modal data, such as patient health records, miRNA and other multi-omics data [5], ultrasound [46], mammography [47], and other novel imaging methods [48,49,50]. Finally, we aim to evaluate our proposed model across various anatomical locations, physiological conditions, and imaging modalities to assess its broad applicability and effectiveness in diverse clinical settings.

**Table 3:** Comparison of different learning methods with our proposed self-contrastive method for breast MWR data.

Method	Reference	Description	Comparison
Supervised Learning	[51,52,53]	Trains models by learning features that map inputs to predefined labels from annotated datasets.	Highly effective for breast cancer detection but requires large, diverse, and clean datasets. The proposed self-contrastive learning reduces data dependency by focusing on feature extraction through comparisons within the sample.
Unsupervised Learning	[51,54,55]	Learns patterns and structures from data without labeled supervision, often using clustering or autoencoders.	Does not rely on labeled data, making it useful for feature discovery but may have reduced performance. In contrast, our self-contrastive approach is reliant on the labeled data to improve feature learning through local/regional instance discrimination.
Transfer Learning	[39,56,57,58,59]	Utilizes pre-trained models from other domains or imaging modalities, fine-tuning them for breast cancer detection.	Improves accuracy, reduces training time, and requires fewer labeled samples. However, performance depends on domain similarity between the pre-trained and target datasets. Self-contrastive learning, in contrast, learns from the target dataset directly.
Contrastive Learning	[39,40,41,42]	Combines multiple models (e.g., random forests, boosting, bagging, neural networks) to enhance classification accuracy.	Increases robustness and generalization but requires more computational resources and diverse model architectures. Self-contrastive learning used here resembles ensemble learning, however, requires manual definition of the individual models to obtain the desirable diverse models.
Ensemble Learning	[60,61,62]	Combines multiple models (e.g., random forests, boosting, bagging, neural networks) to enhance classification accuracy.	Increases robustness and generalization but requires more computational resources and diverse model architectures. Self-contrastive learning used here resembles ensemble learning, however, requires manual definition of the individual models to obtain the desirable diverse models.
Explainable AI	[63,64,65,66,67]	Improves model interpretability by providing explanations for predictions by either indicating which regions contributed to the final result or by presenting similar samples in the training set.	Helps clinicians understand model decisions but may introduce complexity in implementation. Self-contrastive learning primarily focuses on feature representation rather than interpretability.

## 5. Conclusions

Our research introduces promising advancements in MWR-based breast cancer detection, highlighting the substantial potential for clinical applications and future exploration. The proposed models, particularly J-MWR, demonstrate robust performance in accurately identifying breast cancer with high generalizability and reliability. By combining thermal asymmetry analysis with data-driven methodologies, we bridge the gap between traditional diagnostic approaches and modern deep learning techniques, paving the way for more efficient and accurate breast cancer detection. The integration of J-MWR into the MWR-2020 device for point-of-care testing can potentially alleviate the need for training clinical staff to interpret temperature readings, enabling faster and more accurate decision-making. While challenges remain, particularly in ensuring the models’ applicability across all patient demographics, future work will focus on addressing these barriers and advancing the technology for broader and more equitable use.

## Figures and Tables

**Table 1:** The mean and standard deviation of Matthews correlation coefficient (MCC), accuracy, and receiver operating characteristic (ROC) area under the curve (AUC) for the base model and the proposed models: L-MWR, R-MWR, G-MWR, and J-MWR. Values in **bold** indicate the best performance.

Model	MCC	Accuracy	ROC AUC
Base	0.58 ± 0.004	0.88 *±* 0.003	0.93 *±* 0.006
L-MWR	0.43 *±* 0.002	0.82 *±* 0.002	0.89 *±* 0.001
R-MWR	0.66 *±* 0.012	0.92 *±* 0.003	0.95 *±* 0.006
G-MWR	0.61 *±* 0.045	0.90 *±* 0.021	0.94 *±* 0.016
J-MWR	**0.74 *±* 0.018**	**0.95 *±* 0.003**	**0.96 *±* 0.001**

**Table 2:** The mean and standard deviation of Matthews correlation coefficient (MCC) results for each model trained with batch-wise contrastive losses (contrastive, N-pairs, triplet hard, and triplet semi-hard). MCC values in **bold** indicate improvements over their non-batch-wise loss counterparts.

Model	MCC			
	Contrastive	N-Pairs	Triplet Hard	Triplet Semi-Hard
Base	**0.60 *±* 0.024**	**0.60 ± 0.035**	0.57 *±* 0.016	0.51 *±* 0.149
L-MWR	**0.44 *±* 0.003**	**0.44 *±* 0.001**	**0.44 *±* 0.004**	**0.44 *±* 0.008**
R-MWR	0.63 *±* 0.060	0.59 *±* 0.019	0.60 *±* 0.026	0.64 *±* 0.025
G-MWR	**0.65 *±* 0.005**	**0.64 *±* 0.012**	**0.64 *±* 0.022**	**0.67 *±* 0.017**
J-MWR	0.71 *±* 0.000	0.71 *±* 0.029	0.74 *±* 0.030	0.70 *±* 0.081

## Data Availability

Data are available upon request. The source code is available at https://github.com/cgalaz01/self_contrastive_mwr (accessed on 1 February 2025).
